# Longitudinal study of the relationship between coffee consumption and type 2 diabetes in Chinese adult residents: Data from China Health and Nutrition Survey

**DOI:** 10.1371/journal.pone.0251377

**Published:** 2021-05-10

**Authors:** Zhenkai Ma, Mo Hao

**Affiliations:** 1 Research Institute of Health Development Strategies, Fudan University, Shanghai, China; 2 Collaborative Innovation Center of Social Risks Governance in Health, Fudan University, Shanghai, China; University of Mississippi Medical Center, UNITED STATES

## Abstract

**Background:**

Increasing coffee intake was inversely associated with risk of type 2 diabetes in Western countries. However, in China where coffee consumption and diabetes population has been growing fast in recent years, studies on the impact of coffee intakes on the onset of type 2 diabetes are lacking. This study attempts to determine the associations between coffee consumption and type 2 diabetes in Chinese adults.

**Methods:**

This longitudinal study analyzed 10447 adults who had participated in at least two rounds of the China Health and Nutrition Survey (CHNS), which is a survey database of multistage, random cluster process during 1993–2011. Coffee consumption and type 2 diabetes incidence were measured in the survey. Body mass index (BMI), age, sex, place of residence, waves, education level, smoking, drinking alcohol and tea drinking frequency were adjusted as covariate. We used longitudinal fixed effects regression models to assess changes within person.

**Results:**

After adjusting confounding factors, lower risk of diabetes is observed among Chinese adults who drink coffee occasionally (Adjusted Odds Ratio (AOR)  = 0.13, 95% CI = 0.05, 0.34) and drink almost every day (AOR = 0.61, 95% CI = 0.45, 0.83), compared with those who do not or hardly drink. In the subgroup analysis, among women aged 45–59 who drink coffee one to three times a week (AOR = 0.21, 95% CI = 0.08, 0.52) and men over 60 who drink coffee almost every day (AOR = 0.19, 95% CI = 0.07, 0.53), protective effects were found. For young men aged 19–29, drinking coffee almost every day showed a risk effect (AOR = 20.21, 95% CI = 5.96–68.57).

**Conclusions:**

Coffee drinking habit is an independent protective factor for adult on type 2 diabetes in China. And it varies among people with different ages and genders. The rapid growth of coffee consumption in China in recent years may help reduce the risk of type 2 diabetes, but at the same time, the risk of type 2 diabetes in adolescents needs attention.

## Introduction

Type 2 diabetes (T2D) is the most common form of diabetes. It is a complex endocrine metabolic disease associated with eating habits and is considered to be a major health care problem in China at present [1], which has brought a heavy financial burden [2]. T2D is caused by multiple factors, while coffee consumption is well known to be inversely associated with T2D [3]. China as a country with long history of drinking green tea, the demand for coffee has also grown rapidly in recent years [4]. However, research on the incidence of T2D and coffee consumption is lacking in China.

Compared to people who do not or hardly drink coffee, some health benefits and lower risk of chronic disease has been observed on people consuming 3–4 cups per day [5]. Caffeine in coffee has been proved with effect to improve insulin sensitivity [6]. Studies about varies types of coffee can contribute to diabetes prevention [7–10] have been inquired, but not until recently has it been proved that coffee is also related to prevention of T2D [3,11–14]. Some systematic analysis [15,16] supports the hypothesis that habitual coffee consumption is associated with lower risk of T2D.

However, most of previous findings on the relationship between coffee consumption and health have been conducted in Western countries and among populations of European descent [17]. This longitudinal study aims to investigate the relationship between coffee consumption and T2D in China.

## Methods

### Study population

The research data comes from the CHNS [18], a public database which does not involve Human Subject Research, Animal Research and Field Research. The data has been anonymized to make sure no personal private information contained. Instead, a sequence of unique id was used in this study. All participants provided informed consent and the study has been approved by institutional review board from the University of North Carolina at Chapel Hill, the National Institute for Nutrition and Food Safety and China Center for Disease Control and Prevention.

The CHNS is an ongoing, large-scale, longitudinal, household-based survey to investigate the health and nutritional status of the general population in China. The prospective household-based study surveyed nine provinces: Heilongjiang, Liaoning, Shandong, Jiangsu, Henan, Hubei, Hunan, Guangxi, and Guizhou. Provinces with diverse demography, geography, economic development, and public resource characteristics were surveyed by a multistage, random cluster process. The CHNS and the survey procedure has been described in detail elsewhere [18,19]. Ten rounds of surveys were completed between 1989 and 2015. This study selected Chinese residents aged between 18 and 80 years who participated in the surveys from 1993 to 2011 as longitudinal tracking people, excluding pregnant women, lactating mothers, and subjects with incomplete records of key analysis variables. Ultimately, 54,645 observations containing 10,447 adults who participated in at least 2 rounds of the survey were chosen as study subjects.

## Data quality assurance

As the information provided by documents on CHNS official website http://www.cpc.unc.edu/projects/china/data/data.html, several data quality assurance methods were used. Trainings and field guides were distributed to data collectors and supervisors. Questionnaires were checked for consistency and completeness by supervisors at the end of every day. According to the unique id of each person, required data collections from different rounds were merged by using scripts of R language (version 4.0.3). Following data de-duplication and cleaning are mainly carried out using the R package data.table (version: 1.14.0). The whole process of data combination, cleaning, and analyzing was performed in a unified environment of R to ensure that every step of the data processing is fully recorded and can be reproduced.

## Operational definition

**Type 2 diabetes** status was identified by the following question during the follow-up survey: “Has a doctor ever told you that you have diabetes?” If yes, “How old were you when you were told about the situation (diabetes)?” In the 2009 interview, blood sample of participants were collected in addition to the self-determine question in the survey, with which fasting plasma glucose and glycated hemoglobin (HbA1c) were checked. According to the 2010 American Diabetes Association criteria [20], participants with a fasting plasma glucose ≥ 7.0 mmol/L or HbA1c ≥ 6.5% were diagnosed as diabetes patients. Both self-reported and plasma glucose/HbA1c determined T2D cases were included [21].

In each questionnaire, participants were asked how often on average had they consumed coffee. The participants could choose from 9 responses of drinking frequency from drinking every day to 30 days without drinking. The coffee drinking behavior was divided into three groups: no or hardly drink, one to three times a week, drink almost every day.

## Statistical analysis

R was used for all data collation and basic statistical analysis. R package lme4 (version 1.1–23) was used to establish longitudinal fixed effects regression models to explore the crude and adjusted fixed effects between coffee drinking frequency and T2D. The individual itself and the group composed of sequences in multiple observations formed up two-layer model. Three models were evaluated:1) unadjusted crude associations were first examined, and 2) then these associations were adjusted for tea drinking frequency, BMI, age, waves, marital status, education level and place of residence. 3) Model 3 added smoking, drinking alcohol for further discussion.

Odds ratios (ORs) with 95% confidence intervals (CIs) were presented to show the strength and direction of the association. The criterion for statistical significance was set at p < = 0.05. The variable assignment is listed in Table 1. The place of residence was divided into urban and rural. There were two types of marital status: unmarried or married. The education level was divided into four categories: primary school and below, junior high school, senior high school, and college and above. BMI and age are both treated as continuous variables. Smoking and drinking are classified as whether there is such a habit.

**Table 1:** The classification of model variables.

Variable	Classification	Sorting Classification
Age(year)	Categorical variables	18–29 = 1, 30–44 = 2, 45–59 = 3, > = 60 = 4, reference is 1
Place of residence	Categorical variables	Urban = 1, rural = 0
Gender	Categorical variables	Male = 1, female = 0
Marital status	Categorical variables	Married = 1, Unmarried = 0
Education level	Categorical variables	Primary school and below = 0, junior and senior high school = 1, college and above = 2
BMI	Continuous variable	
Waves	Categorical variables	All waves (1993, 1997, 2000, 2004, 2006, 2009 and 2011) are treated as dummy variables valued by 0 and 1
Smoking	Categorical variables	No = 0, yes = 1
Drinking alcohol	Categorical variables	No = 0, yes = 1
Type 2 diabetes	Categorical variables	No = 0, yes = 1
Coffee	Categorical variables	No or hardly drink = 0, one to three times a week = 1, drink almost every day = 2

## Results

### Demographic characteristics

Table 2 presented the demographic characteristics of the adult participants in the CHNS during 1993–2011 whose complete data were available. The first round of the CHNS, including individual, household, community, and health/family planning facility data, was collected in 1989. Eight additional panels were collected in 1991, 1993, 1997, 2000, 2004, 2006, 2009 and 2011 [22]. Since the 1993 survey, all new households formed from sample households were added. There was a gradual growth in the coffee drinking frequency during the seven waves, and the prevalence of T2D also showed an upward trend, from 7.9% in 1993 to 10.0% in 2011. Regarding to obesity and aging, which is most closely related to the onset of T2D, we can see that BMI increased from 22.5 in 1993 to 24.6 in 2011, and it has almost reached the normal standard of 24.9 recommended by the WHO [23]. People over 60 years old increased from 10.3% in 1993 to 23.6% in 2011, a more than two-fold increase.

**Table 2:** Demographic characteristics of participants.

Variables	Survey Year						
	1993	1997	2000	2004	2006	2009	2011
Total (N)	3413	4457	8589	8245	8042	8620	13279
Gender (%)							
Female	1771 (51.9)	2185 (49)	4338 (50.5)	425 3(51.6)	4182 (52)	4418 (51.3)	6883 (51.8)
Male	1642 (48.1)	2272 (51)	4251 (49.5)	3992 (48.4)	3860 (48)	4202 (48.7)	6396 (48.2)
Age (%)							
18–30	834 (24.4)	1531 (34.4)	1724 (20.1)	978 (11.9)	693 (8.6)	861 (10)	1331 (10)
31–44	1590 (46.6)	1700 (38.1)	3662 (42.6)	3153 (38.2)	2998 (37.3)	2454 (28.5)	3728 (28.1)
45–59	639 (18.7)	924 (20.7)	2266 (26.4)	2746 (33.3)	2761 (34.3)	3388 (39.3)	5083 (38.3)
60–80	350 (10.3)	302 (6.8)	937 (10.9)	1368 (16.6)	1590 (19.8)	1917 (22.2)	3137 (23.6)
Marital status (%)							
Unmarried	482 (14.1)	670 (15)	1249 (14.5)	1109 (13.5)	891 (11.1)	1103 (12.8)	1749 (13.2)
Married	2931 (85.9)	3787 (85)	7340 (85.5)	7136 (86.5)	7151 (88.9)	7517 (87.2)	11530 (86.8)
Education level (%)							
Primary school and below	1223 (35.8)	2049 (46)	3019 (35.1)	3004 (36.4)	2808 (34.9)	3158 (36.6)	3171 (23.9)
Junior and senior high school	1847 (54.1)	1744 (39.1)	4127 (48)	3845 (46.6)	3713 (46.2)	3905 (45.3)	5929 (44.6)
College and above	343 (10)	664 (14.9)	1443 (16.8)	1396 (16.9)	1521 (18.9)	1557 (18.1)	4179 (31.5)
Place of residence							
Rural	2123 (62.2)	2704 (60.7)	5775 (67.2)	5412 (65.6)	5270 (65.5)	5810 (67.4)	7267 (54.7)
Urban	1290 (37.8)	1753 (39.3)	2814 (32.8)	2833 (34.4)	2772 (34.5)	2810 (32.6)	6012 (45.3)
Drinking alcohol							
No	2166 (63.5)	2570 (57.7)	5712 (66.5)	5485 (66.5)	5344 (66.5)	5711 (66.3)	8492 (64)
Yes	1247 (36.5)	1887 (42.3)	2877 (33.5)	2760 (33.5)	2698 (33.5)	2909 (33.7)	4787 (36)
Smoking							
No	2267 (66.4)	2874 (64.5)	5864 (68.3)	5666 (68.7)	5658 (70.4)	6083 (70.6)	9746 (73.4)
Yes	1146 (33.6)	1583 (35.5)	2725 (31.7)	2579 (31.3)	2384 (29.6)	2537 (29.4)	3533 (26.6)
Coffee frequency							
No or hardly drink	3379 (99)	4393 (98.6)	8512 (99.1)	8160 (99)	7903 (98.3)	8358 (97)	12054 (90.8)
One to three times a week	7 (0.2)	23 (0.5)	30 (0.3)	18 (0.2)	20 (0.2)	41 (0.5)	238 (1.8)
Drink almost every day	27 (0.8)	41 (0.9)	47 (0.5)	67 (0.8)	119 (1.5)	221 (2.6)	987 (7.4)
Type 2 diabetes							
No	3144 (92.1)	4170 (93.6)	7923 (92.2)	7475 (90.7)	7231 (89.9)	7647 (88.7)	11953 (90)
Yes	269 (7.9)	287 (6.4)	666 (7.8)	770 (9.3)	811 (10.1)	973 (11.3)	1326 (10)
BMI							
Mean	22.5	22.7	23.5	23.7	23.8	23.7	24.6
Median	22.1	22.2	23.2	23.3	23.4	23.5	24.2
Standard deviation	2.9	3.1	3.4	3.5	3.4	3.5	4.6

### Association between coffee and type 2 diabetes

The crude analysis showed that compared with participants who do not or hardly drink a cup of coffee per month, for those who drink one to three times a week and more frequently, the odds of T2D are 87% and 50% lower (Crude Odds Ratio (COR) = 0.13, 95% CI = 0.05–0.31 and COR = 0.5, 95% CI = 0.4–0.64).

After adjusting for confounding factors including tea drinking frequency, BMI, age, waves, marital status, education level and place of residence, the one to three times a week drinking behavior still exerted a statistically significant effect on T2D (Adjusted Odds Ratio (AOR) = 0.13, 95% CI = 0.05–0.34). Meanwhile, the inverse relationship (AOR = 0.6, 95% CI = 0.47–0.81) of drink almost every day is slightly smaller than the former.

The specific results are presented in Table 3, which indicates that aging (Over 60 years old, AOR = 13.86, 95% CI = 11.32–16.98) and obesity (BMI, AOR = 1.13, 95% CI = 1.12–1.14) were risk factors for T2D. For those who have received college education or above, education level is a protective factor (AOR = 0.88, 95% CI = 0.8–0.97) on T2D. Compared with women, men are at greater risk (AOR = 1.07, 95% CI = 1–1.14). Drinking tea almost every day has similar protective effect as coffee (AOR = 0.91, 95% CI = 0.84–0.98). However, the habit of drinking tea one to three times a week is not statistically significant.

**Table 3:** Results of fixed effects regression analysis investigating the association of coffee consumption with the risk of type 2 diabetes among Chinese adults (1993–2011).

Independent variables:	Dependent variable: type 2 diabetes		
	Model 1	Model 2	Model 3
Coffee frequency (ref = No or hardly drink)			
one to three times a week	0.13 (0.05, 0.31)***	0.13 (0.05, 0.34)***	0.13 (0.05, 0.34)***
drink almost every day	0.5 (0.4, 0.64)***	0.6 (0.45, 0.81)***	0.61 (0.45, 0.83)**
Tea frequency (ref = No or hardly drink)			
one to three times a week		1.05 (0.85, 1.29)	1.07 (0.87, 1.32)
drink almost every day		0.91 (0.84, 0.98)*	0.93 (0.86, 1.01)
BMI		1.13 (1.12, 1.14)***	1.13 (1.13, 1.14)***
Age (ref = 18–29)			
30–44		3.63 (2.96, 4.44)***	3.7 (3.02, 4.53)***
45–59		7.77 (6.36, 9.5)***	7.9 (6.47, 9.66)***
> = 60		13.86*** (11.32, 16.98)	13.95 (11.39, 17.08)***
Sex (ref = Female)		1.07 (1, 1.14)*	1.15 (1.07, 1.24)***
Time (ref = 1993)			
1997		0.85 (0.71, 1.02)	0.86 (0.72, 1.03)
2000		0.77 (0.66, 0.89)***	0.76 (0.65, 0.88)***
2004		0.75 (0.65, 0.87)***	0.74 (0.64, 0.86)***
2006		0.77 (0.67, 0.9)***	0.76 (0.66, 0.89)***
2009		0.82 (0.7, 0.95)**	0.81 (0.7, 0.94)**
2011		0.65 (0.56, 0.75)***	0.64 (0.55, 0.74)***
Marital status (ref = Unmarried)		1.07 (0.96, 1.19)	1.08 (0.97, 1.19)
Education level (ref = Primary school and below)			
Junior and senior high school		0.96 (0.9, 1.03)	0.98 (0.91, 1.05)
College and above		0.88 (0.8, 0.97)**	0.91 (0.83, 0.99)*
Place of residence (ref = Rural))		0.95 (0.89, 1.02)	0.96 (0.9, 1.02)
Smoking (ref = No))			1.09 (1.01, 1.17)*
Drinking alcohol (ref = No))			0.79 (0.73, 0.86)***

Note: *p<0.05

**p<0.01

***p<0.001.

After further added other addictive common factors which is the habit of smoking and drinking alcohol, the statistical significance of coffee still stand and the correlation coefficient almost remains unchanged. The statistical significance of tea drinking frequency disappeared. This shows that the influence of tea drinking behavior on T2D might relate to other factors of lifestyle. As expected, smoking is a risk factor for T2D (AOR = 1.09, 95% CI = 1.01–1.17). However, on contrary to popular belief, drinking is a protective factor for T2D (AOR = 0.79, 95% CI = 0.73–0.86).

The analysis based on different gender and age group implies the protective effect varies among people in Table 4. Among middle-aged (45–59 years old) women with a habit of drinking coffee almost every day, protective effect for T2D was observed (AOR = 0.21, 95% CI = 0.08–0.52). Same protective effect (AOR = 0.19, 95% CI = 0.07–0.53) also appears in elderly (> = 60 years old) men who drinks one to three times a week. But among young men (18–29 years old) with the habit of drinking coffee regularly, negative effect has been observed (AOR = 20.21, 95% CI = 5.96–68.57).

**Table 4:** Association of coffee consumption with the risk of type 2 diabetes by gender and age subgroup.

age	Variable		
	Coffee frequency (ref = No or hardly drink)	Males	Females
18–29	One to three times a week	——	——
	Drink almost every day	20.21 (5.96, 68.57)***	——
30–44	One to three times a week	——	——
	Drink almost every day	0.70 (0.37, 1.33)	——
45–59	One to three times a week	——	0.21 (0.08, 0.52)***
	Drink almost every day	1.17 (0.71, 1.92)	——
> = 60	One to three times a week	2.32 (0.74, 7.22)	——
	Drink almost every day	0.19 (0.07, 0.53)**	0.59 (0.14, 2.58)

Note: *p<0.05

**p<0.01

***p<0.001;——p>0.9.

Each group is investigated by a multilevel logistic regression to analyze the association between coffee consumption and the risk of type 2 diabetes, including coffee drinking frequency, tea drinking frequency, BMI, waves, marital status, education level, smoking, drinking alcohol and place of residence. The regression coefficients with p value greater than 0.9 are omitted.

## Discussion

This longitudinal study explored the association between coffee consumption and the prevalence of T2D in Chinese adults. The single-factor model of Model 1 shows that coffee consumption has a crude reverse correlation effect on T2D. The multifactor analysis includes drinking tea, BMI, sex, age, place of residence, waves, marital status and education level as covariates. Model 2 for all adults suggested that drinking coffee is a protective factor for T2D in China. We did not observe stronger protective effect on people who drink it almost every day than people who drink one to three times a week.

The protective effect of drinking tea on T2D disappeared after smoking and drinking were added in Model 3. This may be related to more complicated lifestyle habits, which remains to be further studied and clarified. The protective effect of drinking alcohol on diabetes is consistent with some evidence [27,28] that a small amount of alcohol has positive health effects. Since China has a long-term drinking culture, adult drinking is a quite common phenomenon. The question of whether if you drink alcohol in the questionnaire may not be able to accurately measure their drinking habits. People who answered yes probably only drink very little in their daily lives.

The models of subdivided participants revealed that the effect of coffee on T2D varies among genders and ages. For middle-aged women and elderly men, the habit of drinking coffee is a protective effect on T2D. However, among young men, those who drink coffee regularly are more likely to have T2D than those who hardly drink coffee. The impact of coffee on T2D in Chinese young people, especially young men, still requires further research.

The reported association between coffee and T2D has not been entirely consistent across different countries and ethnicities. Studies performed in Dutch [24], Sweden [25], Spain [14], Netherlands [26], Japan [27], and the United States [14] found that coffee consumption reduces the risk of T2D, which is consistent with the results of our study. However, some studies have shown that coffee consumption in adults and adolescents has a U-shaped relationship with T2D, which means that excessive coffee drinking will bring potential health risks [28–30]. In present study we did not observe a U-shaped relationship between coffee and the prevalence of T2D, moreover, drinking coffee almost every day did not reduce more risk on T2D in China. Possible causes for these discrepancies include differences in race, sample size, average age of the population, correction factors, and differences in the grouping of coffee consumption.

Many studies have shown that acute caffeine ingestion reduces insulin sensitivity [31]. This study found a habit of drinking coffee to be an independent protective factor for T2D in China, which might be due to the following mechanisms:

1. Coffee consumption is associated with widespread metabolic changes, among which lipid metabolites may be critical for the anti-diabetes benefit of coffee. Coffee-related metabolites might help improve prediction of diabetes [32].
2. Coffee interferes with glucose homeostasis, Long-term consumption of both coffee species reduced weight gain and liver steatosis and improved insulin sensitivity in the model of T2D [33].
3. Chlorogenic acids may affect glucose absorption and subsequent utilization, the latter through metabolites derived from endogenous pathways or action of the gut microbiota [34,35].

The strength of this study is the largeness of sample, which means that the results of the multivariate longitudinal fixed effects regression analyses is stable. However, compared with related research performed in other countries, this study is limited by the contents of the CHNS questionnaire, and it does not consider other relevant information related to coffee consumption such as the type of coffee. Some studies have considered the interaction between coffee consumption and types of coffee and found that the protective effect of coffee on T2D is quite different by different kinds of coffee [36]. In addition, conclusions of this paper mainly come from the subjective report of the questionnaire and a biometric measurement, which may neglect the potential diabetic population in the long-term process.

In summary, a Coffee drinking habit is an independent protective factor for adult T2D. And it varies among people of different ages and genders. The rapid growth of coffee consumption in China in recent years may help reduce the risk of type 2 diabetes, but at the same time, the risk of type 2 diabetes in adolescents requires attention.
